# Comparisons of Non-Oral Immune-Related Adverse Events Among Patients With Cancer With Different Oral Toxicity Profiles

**DOI:** 10.1093/oncolo/oyad279

**Published:** 2023-10-24

**Authors:** Yuanming Xu, Natalie Wen, Robert I Haddad, Stephen T Sonis, Alessandro Villa

**Affiliations:** Department of Diagnostic Sciences, Tufts University School of Dental Medicine, Boston, MA, USA; Department of Oral Medicine, Infection, and Immunity, Havard School of Dental Medicine, Boston, MA, USA; Division of Oral Medicine and Dentistry, Brigham and Women’s Hospital, Boston, MA, USA; Department of Oral Medicine, Infection, and Immunity, Havard School of Dental Medicine, Boston, MA, USA; Department of Medical Oncology, Dana-Farber Cancer Institute, Boston, MA, USA; Department of Oral Medicine, Infection, and Immunity, Havard School of Dental Medicine, Boston, MA, USA; Division of Oral Medicine and Dentistry, Brigham and Women’s Hospital, Boston, MA, USA; Oral Medicine and Oral Oncology, Miami Cancer Institute, Miami, FL, USA; Department of Orofacial Sciences, University of California San Francisco, San Francisco, CA, USA

**Keywords:** immune checkpoint inhibitor therapy, immune-related adverse events, oral mucositis, xerostomia, dysgeusia, melanoma, lung neoplasms, head and neck cancer

## Abstract

**Objectives:**

Immune-related adverse events (irAEs) are common. Oral irAEs tend to cluster in patients who experience concurrent toxicities. We aimed to characterize the frequency and trajectory of non-oral irAEs in patients who developed oral irAEs, assess their relationship with non-oral irAEs, and compare those characteristics with patients without oral irAEs.

**Methods:**

A retrospective chart review was conducted to identify patients who started ICIT between December 11, 2011, and September 15, 2019 (*n* = 4683) in the Mass General Brigham Registered Patient Data Registry. Demographic information, cancer diagnosis, ICIT regimen, treatment duration, and time and number of infusions to irAE onset were recorded. Non-oral irAEs were categorized into 13 groups. Patients with melanoma, pulmonary cancer, or head and neck cancer who had oral irAEs were then matched with those without oral irAEs to compare the prevalence of concomitant non-oral irAEs.

**Results:**

Three hundred and fourteen patients with oral irAEs with a mean age of 65.9 ± 12.6 years (43.3% females) were included. Patients with multiple oral irAEs were more likely to have non-oral irAEs (OR: 2.7, 95% CI, 1.3-3.5), including cutaneous (OR: 1.7, 95% CI, 1.1-3.0), rheumatological (OR: 2.2, 95% CI, 1.1-4.2), thyroid (OR: 2.4, 95% CI, 1.2-4.9), and neurological irAEs (OR: 2.5, 95% CI, 1.0-6.3). Compared to matched patients with non-oral irAEs, patients with oral irAEs were more likely to have cutaneous (OR: 1.7, 95% CI, 1.0-2.8) and thyroid (OR: 2.86, 95% CI, 1.1-7.5) irAEs. The development of oral and non-oral irAEs is often coincidental.

**Conclusion:**

Patients who have non-oral irAEs should be monitored for development of oral irAEs for prompt management.

## Teaser

This article characterizes the frequency and trajectory of nonoral immune checkpoint inhibitor treatment-related toxicities in patients who developed oral immune-related adverse events (irAEs) and compares those characteristics with patients without oral irAEs.

**Implications for Practice**

Results of this study indicate that patients who develop multiple oral immune-related adverse events (irAEs) are more likely to develop non-oral irAEs, specifically cutaneous, gastrointestinal, and rheumatological irAEs. Oral and non-oral irAEs follow similar trajectories, with the onset of non-oral irAEs occurring before oral irAEs. These results provide insight on the relationship of oral irAEs to other irAEs and may be used for management of irAEs throughout cancer treatment.

## Introduction

Immune checkpoint inhibitors (ICIs) are a class of immunotherapy drugs that have revolutionized cancer treatment by targeting membrane protein programmed cell death protein 1 (PD-1), programmed cell death protein ligand 1 (PD-L1), and cytotoxic T-lymphocyte associated protein 4 (CTLA-4) and blocking the inhibitory signal on the T cells, thus reactivating both innate and adaptive immune systems against cancer cells.^1^

The enhanced immune response targeting the cancer cells can also induce inflammatory or autoimmune-like side effects against any organ due to the release of proinflammatory cytokines, complement activation, antibody, or T-cell-mediated immune-inflammatory response.^2,3^ Immune-related adverse events (irAEs) (all grades) are common in patients with cancer treated with ICIs, with an incidence of 72% in patients receiving CTLA-4 inhibitor,^4,5^ 66% in patients treated with PD-1/PD-L1 inhibitors,^5^ and 87% in patients with combined immunotherapy.^6^ Severe irAEs (≥grade 3) were reported in 14%, 34%, and 55% of patients treated with PD-1/PD-L1 inhibitors, CTLA-4 inhibitors, and ICIs combinations, respectively.^7^ Dermatologic, gastroenterological, hepatic, endocrine, respiratory, and rheumatologic (musculoskeletal) side effects are most commonly reported, with onsets varying from weeks to months after the ICI initiation and may reflect the variations in pathogenesis.^3^ Differences in irAE prevalence and trajectory are associated with the affected organ system and immunotherapy regimen. Interestingly, cancer treatment outcomes are also associated with irAE patterns.^8^

As previously reported, irAEs affecting the oral cavity and contiguous structures are not infrequent. Oral mucosal disorders, xerostomia, and dysgeusia occur in nearly 7% of patients treated with ICIT.^9,10^ Like toxicities associated with cytotoxic cancer therapies, AEs rarely occur in isolation. Rather, they tend to cluster with patients having simultaneous toxicities, often sharing common pathoetiologies. Defining the nature of these associations has been informative in predicting risk and clinical course. While toxicity clustering has been well described for chemotherapy regimens and radiation, such is not the case with irAEs.

Thus, this large retrospective study aimed to characterize the frequency and trajectory of non-oral ICI treatment-related toxicities in patients who developed oral irAEs and to compare those characteristics with patients without oral irAEs.

## Materials and Methods

### Characteristics of Patients With Oral irAEs

**Figure 1.**
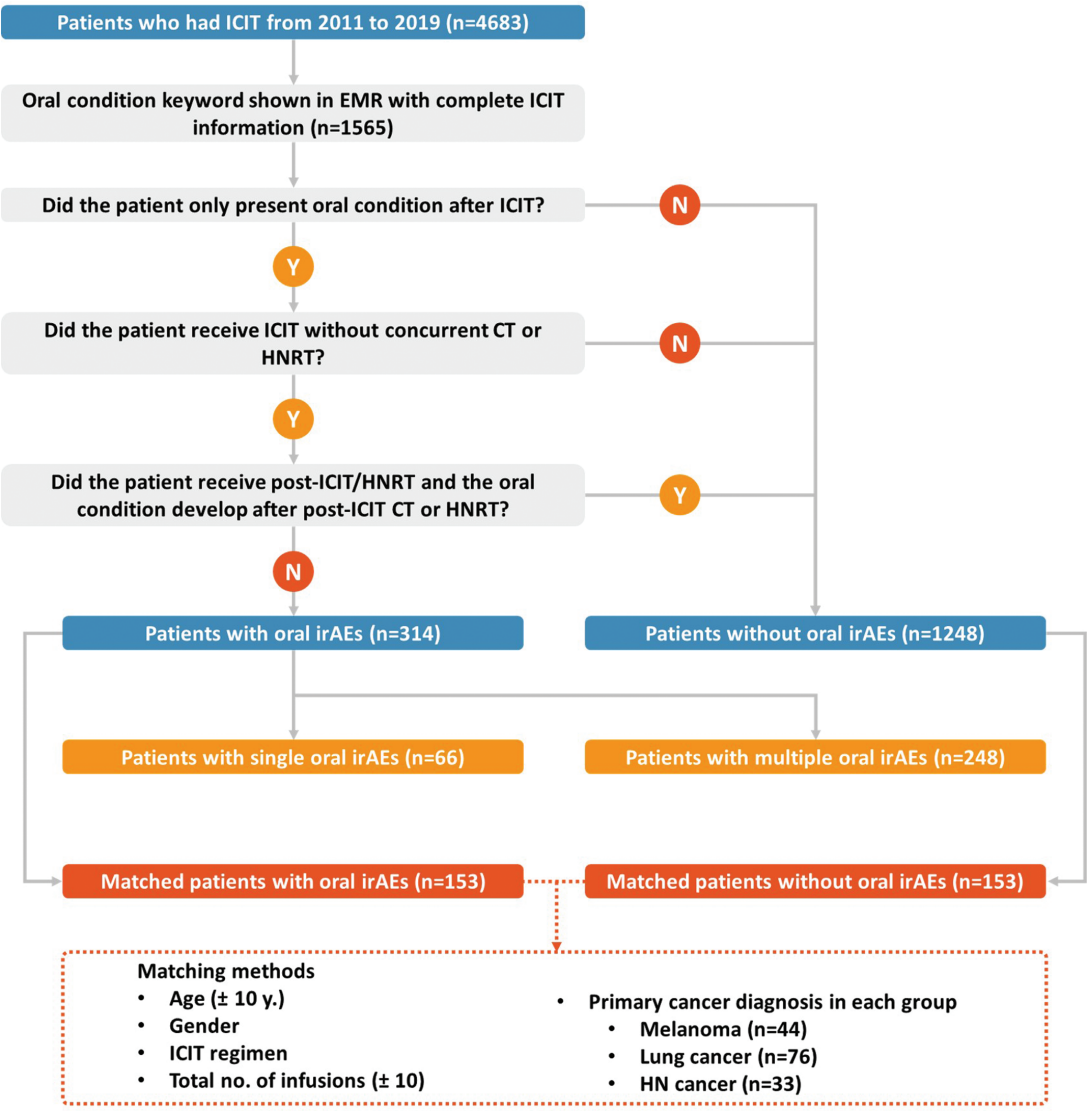
This is a step-by-step illustration of the inclusion of patients with oral irAEs and matched patients without oral irAEs. Of 1565 patients who had oral conditions after ICIT, 314 patients were considered presenting with oral irAEs and 1248 patients without (3 patients were excluded due to conflicting descriptions). In 314 patients with oral irAEs, patients with a single oral irAE were compared with those with multiple oral irAEs. In addition, 153 patients with oral irAEs were matched (1:1 ratio) with patients without oral irAEs for further analysis. Abbreviations: CT: chemotherapy; EMR: electronic medical records; HNRT: head and neck radiation therapy; ICIT: immune checkpoint inhibitor therapy; irAEs: immune-related adverse events.

This study was a continuation of ongoing work on oral irAEs, which has been previously described in detail.^10^ Briefly, a retrospective chart review in the Mass General Brigham Registered Patient Data Registry was conducted to identify patients who started ICIT (nivolumab, pembrolizumab, avelumab, atezolizumab, durvalumab, ipilimumab) between December 11, 2011, and September 15, 2019. Among the 4683 patients who underwent ICIT, the electronic medical records (EMR) of 1565 patients who developed oral conditions after the initiation of ICIT were reviewed. Three hundred and seventeen patients were determined to have developed oral irAEs, including oral mucosal disorders, xerostomia, and dysgeusia. Three patients were excluded from the 317 patients with oral irAEs due to conflicting descriptions of oral symptoms in the EMRs after further review; 314 patients were considered with oral irAEs (Fig. 1).

### Control Group: Patients Without Oral irAEs

From the 1248 patients who did not develop oral irAEs, patients with melanoma, lung cancer, and head and neck cancer were matched to patients with the same type of cancer who developed oral irAEs. Patients were matched based on age (±10 years), gender, type of ICI received, and the total number of ICI infusion doses received (±10 doses).

### Cancer Type and Treatment

Demographic information, the primary cancer diagnosis, the ICIT regimen, the start and end dates of ICIT; the number of ICI infusions before adverse events (AEs) developed; the total number of ICI infusions; and oral irAEs (oral mucosal disorders, xerostomia, and dysgeusia) were reviewed and recorded before September 2021. The time of oral irAE onset relative to ICIT was also recorded.

Non-oral irAEs experienced by patients were identified by reviewing the patients’ EMRs, with primary basis on assessment notes from the oncology teams. The non-oral irAEs were categorized into 13 groups depending on the organ or system affected (cutaneous, gastrointestinal, rheumatological, pulmonary, thyroid, hepatic, neurologic, hematological, renal, ocular, endocrine, pituitary, and cardiac). Due to histological similarity to oral mucosa and salivary glands, we primarily focused on collecting the time of onset and number of ICI infusions before the onset of irAE for cutaneous, gastrointestinal, and pulmonary toxicities. During the review of oncology notes, the onset times of cutaneous, gastrointestinal, and pulmonary irAEs were confirmed by identifying keywords such as rash/pruritus (cutaneous irAEs), diarrhea/colitis (gastrointestinal irAEs), and difficulty in breathing/pneumonitis (pulmonary irAEs), which were first reported in the patient’s progress notes as adverse events related to the ICIT by the oncologist or other clinicians. Data were collected from EMRs and entered in the Mass General Brigham REDCap platform.

### Statistical Analysis

Descriptive statistics were used to describe demographic characteristics and clinical parameters JMP software version 16 (SAS Institute Inc, Cary, NC). The numbers of patients or events for each categorical variable were calculated and presented as percentages. Continuous variables, such as age, the number of infusions, the days of ICIT duration, and the interval in days between the ICIT starting date and the onset of oral or non-oral irAEs, were expressed as means with standard deviations or medians with ranges or interquartile intervals, based on the data distribution. The *t*-test, the Mann-Whitney *U* test, and the Kruskal-Wallis were used to compare differences between continuous variables accordingly. The Wilcoxon signed-rank test was applied for nonparametric paired variables. The associations between the oral irAEs and the non-oral irAEs were determined by the chi-square test and Fisher’s exact test. Logistic regression models were used to estimate odds ratios (ORs) and 95% confidence intervals (CIs) for the correlation between oral irAEs and non-oral irAEs. *P*-values were considered significant at <.05.

## Results

### Characteristics of Patients With Oral irAEs

Three hundred and fourteen patients with oral irAEs were included with a median age of 67 years old (range: 20-91 years); 43.3% patients were females (Table 1). 28.3% (*n* = 89) had lung cancer, 27.1% (*n* = 85) had melanoma, and 14.3% (*n* = 45) had head and neck cancer. 30.3% (*n* = 95) had other cancers including bladder cancer (*n* = 19), esophageal cancer (*n* = 12), liver and gallbladder cancer (*n* = 10), renal cancer (*n* = 10). When ICI was considered, 39.8% of patients (*n* = 125) received pembrolizumab, 32.5% (*n* = 102) nivolumab, and 13.1% (*n* = 41) had combined nivolumab and ipilimumab. 14.6% (*n* = 46) were on other ICIT regimens. Among the 314 patients with oral irAEs, 248 patients had only one oral irAE (155 had xerostomia, 60 had oral mucosal disorders, and 33 had dysgeusia) and 66 patients had more than one oral irAE (Table 1 and Supplementary Material S1).

**Table 1.** Characteristics and non-oral irAEs in patients with oral irAEs.

Characteristics	Patients with only one oral irAE (*n* = 248)	Patients with multiple oral irAEs (*n* = 66)	Total	OR (95% CI)	*P*
Age	66.5 ± 12.6	63.6 ± 12.2	65.9 ± 12.6	—	—
Female (%)	109 (44.0)	27 (40.9)	136 (43.3)	—	—
Most common cancer (%)					
Lung cancer	77 (31.0)	12 (18.2)	89 (28.3)	—	—
Melanoma	54 (21.8)	31 (47.0)	85 (27.1)	—	—
Head and neck cancer	38 (15.3)	7 (10.6)	45 (14.3)	—	—
Immune checkpoint inhibitor regimen (%)					
Pembrolizumab	104 (41.9)	21 (31.8)	125 (39.8)	—	—
Nivolumab	82 (33.1)	20 (30.3)	102 (32.5)	—	—
Ipilimumab + Nivolumab	27 (10.9)	14 (21.2)	41 (13.1)	—	—
Other regimens	35 (14.1)	11 (16.7)	46 (14.6)	—	—
Type of non-oral irAE (%)					
Any	162 (65.3)	55 (83.3)	217 (69.1)	2.7 (1.3-5.3)	.006
Dermatological	87 (35.1)	32 (48.5)	119 (37.9)	1.7 (1.1-3.0)	.047
Gastrointestinal	51 (20.6)	19 (28.8)	70 (22.3)	1.6 (0.9-2.9)	.154
Rheumatological	36 (14.5)	18 (27.3)	54 (17.2)	2.2 (1.1-4.2)	.015
Pulmonary	33 (13.3)	14 (21.2)	47 (15)	1.8 (0.9-3.5)	.110
Thyroid	25 (10.1)	14 (21.2)	39 (12.4)	2.4 (1.2-4.9)	.015
Hepatic	28 (11.3)	4 (6.1)	32 (10.2)	0.6 (0.2-1.5)	.258^a^
Neurologic	13 (5.2)	8 (12.1)	21 (6.7)	2.5 (1.0-6.3)	.047
Hematological	9 (3.6)	4 (6.1)	13 (4.1)	1.7 (0.5-5.8)	.484^a^
Renal	10 (4.0)	3 (4.5)	13 (4.1)	1.2 (0.3-4.2)	.740^a^
Ocular	9 (3.6)	4 (6.1)	13 (4.1)	1.7 (0.5-5.8)	.484^a^
Endocrine	8 (3.2)	2 (3.0)	10 (3.2)	0.9 (0.2-4.5)	1.000^a^
Pituitary	6 (2.4)	1 (1.5)	7 (2.2)	0.6 (0.1-5.3)	1.000^a^
Cardiac	5 (2.0)	2 (3.0)	7 (2.2)	1.5 (0.3-8.0)	.641^a^
Number of non-oral irAEs (IQR)	1 (0, 2)	2 (1, 3)	1 (0, 2)	—	<.001

^a^Fisher’s exact test.

Abbreviations: IQR: interquartile range; irAEs: immune-related adverse events; OR: odds ratio.

### Prevalence and Characteristics of Non-Oral irAEs in Patients With Oral irAEs

Dermatological, gastrointestinal, and rheumatological irAEs were the most common non-oral irAEs with a prevalence of 37.9%, 22.3%, and 17.2%, respectively, followed by pulmonary (15.0%), thyroid (12.4%), and hepatic irAEs (10.2%). Patients with multiple oral irAEs were 2.7 times more likely to have at least one non-oral irAE than those with a single oral irAE (95% CI, 1.3-5.3). Patients with multiple oral irAEs were more likely to have cutaneous (OR: 1.7, 95% CI, 1.1-3.0), rheumatological (OR: 2.2, 95% CI, 1.1-4.2), thyroid (OR: 2.4, 95% CI, 1.2-4.9), and neurological irAEs (OR: 2.5, 95% CI, 1.0-6.3) than those with a single oral irAE (Table 1).

**Figure 2.**
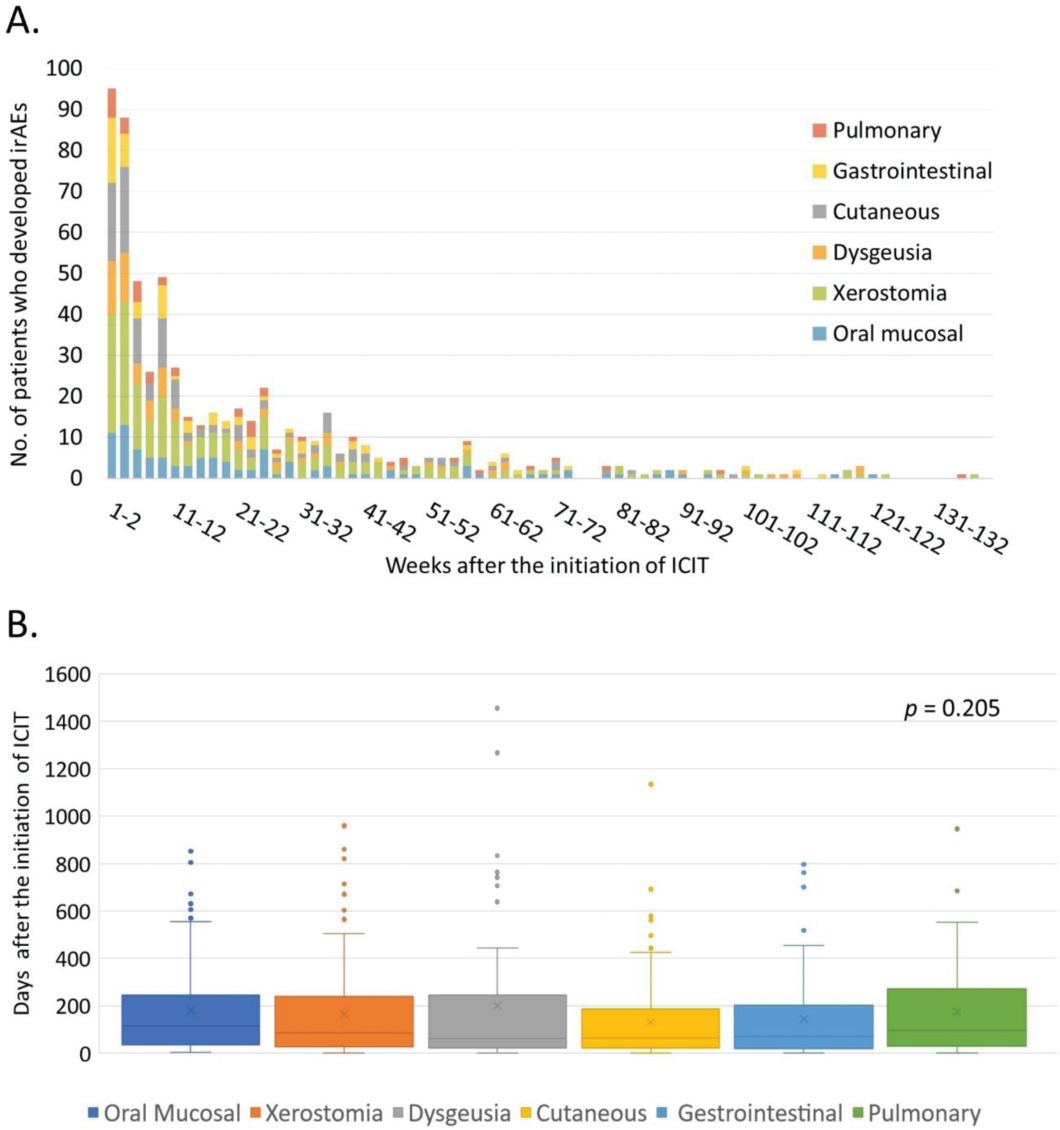
Comparison of the trajectory of the oral and non-oral irAEs in the patients who had oral irAEs. (**A**) Timeline illustrating the number of oral and non-oral immune-related adverse events on a biweekly basis. The histogram shows the distribution of the onset timeline of both oral and non-oral irAEs in the 314 patients who had oral irAEs. We focus on the 3 major non-oral irAEs that affect the skin, the gastrointestinal tract, and the lung. The temporal distribution of non-oral irAE was similar to that of oral irAEs. (**B**) Boxplots show the median, interquartile range, and outlines of the time to the onset of oral and major non-oral irAEs. There is no statistically significant difference among the symptom onset time of the oral and non-oral irAEs (*P* = .205, Kruskal Wallis test). Abbreviations: ICIT: immune checkpoint inhibitor therapy; irAEs: immune-related adverse events.

The temporal distribution of the onsets of oral irAEs and major non-oral irAEs (cutaneous, gastrointestinal, and pulmonary irAEs) is illustrated in Fig. 2A. 65.5% cutaneous, 57.4% gastrointestinal, and 53.2% of pulmonary irAEs developed within 14 weeks (3 months) after the initiation of ICIT. 92.4% cutaneous, 88.6% gastrointestinal, and 83.0% pulmonary irAEs developed within 54 weeks (1 year) after the initiation of ICIT. Among patients who had cutaneous irAEs, the median time to the onset of cutaneous symptoms was 64 days (range: 0-1134 days). Of all patients who had gastrointestinal irAEs, the median time to the onset of gastrointestinal symptoms was 68.5 days (range: 0-796 days). Of all patients who had pulmonary irAEs, the median time to the onset of pulmonary symptoms was 97 (range: 1-946 days). There was no significant difference in the time to onset of these irAEs (Fig. 2B).

**Figure 3.**
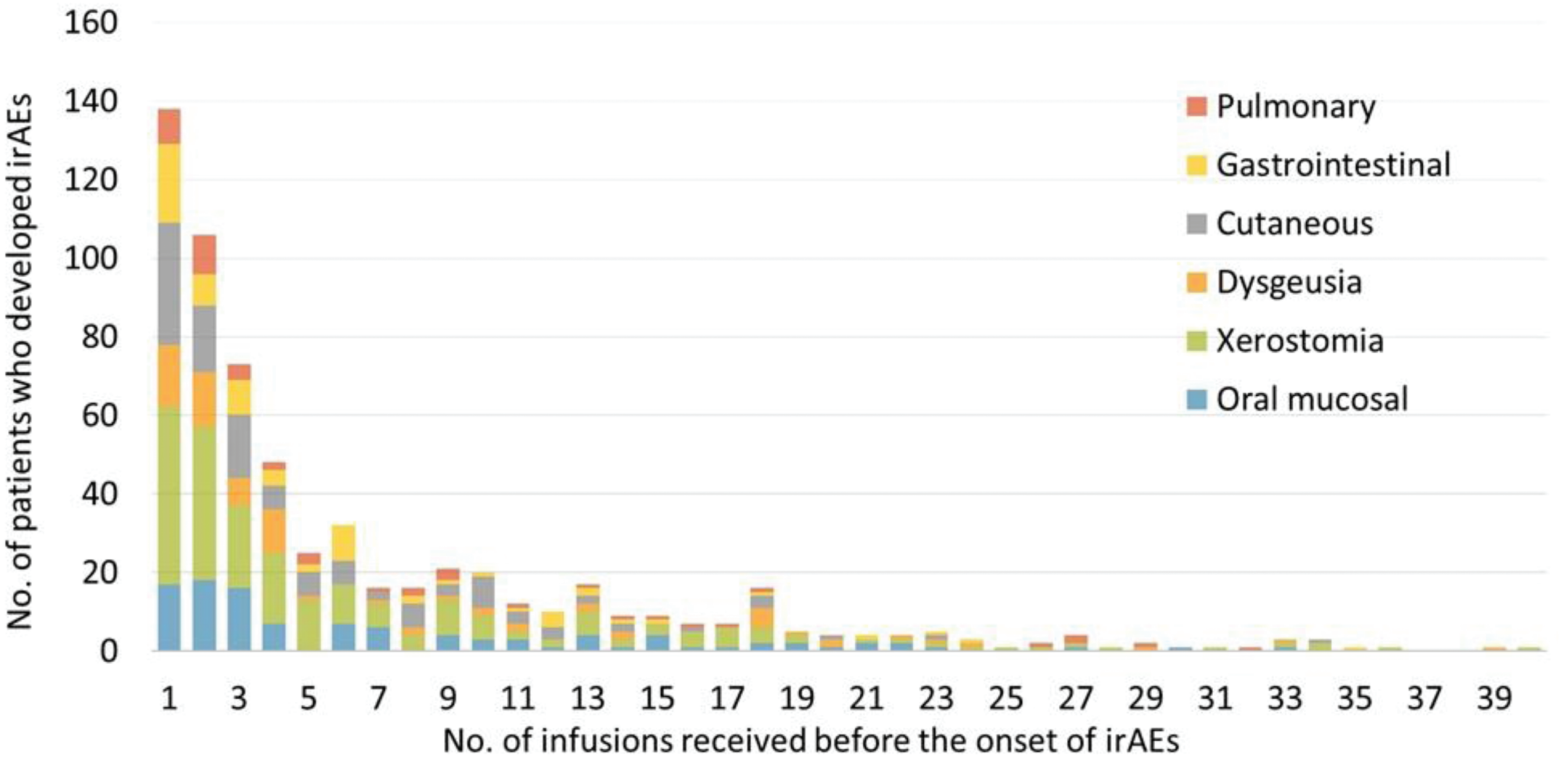
Number of ICIT infusions when patients started to have the onset of oral and non-oral irAEs. The histogram shows a similar distribution of the total number of ICIT infusions by the onset of oral and major non-oral irAEs. Abbreviations: ICIT: immune checkpoint inhibitor therapy; irAEs: immune-related adverse events.

Regarding the relation between the ICIT dosage and the onset timing of the major non-oral irAEs (Fig. 3), the median number of infusions to the onset of cutaneous, gastrointestinal, and pulmonary symptoms was 3 (range from 1 to 62), 3 (range from 1 to 35), and 4 (range from 1 to 41), respectively.

We also compared the onset time of the oral irAEs and major non-oral irAEs in patients who developed both. In patients who presented both oral mucosal and cutaneous irAEs (*n* = 46), cutaneous irAEs occurred with a median day to onset of 42 days (range: 0-691 days) which was significantly shorter than that of 117.5 days (range: 2-671 days) in patients with oral mucosal irAEs (*P* = .04). In patients who had both xerostomia and cutaneous irAEs (*n* = 81), cutaneous irAEs also presented significantly earlier with a median day to onset of 64 days (range: 0-1134 days) than xerostomia with a median day to onset of 144 days (range: 1-959 days). There was no statistically significant difference in the days to onset between other oral and non-oral irAEs in patients who had both (Table 2).

**Table 2.** Comparison of the trajectory of an oral irAE and a non-oral irAE in patients who had both.

n	irAE	Median days to onset (IQR)	*P*
46	Oral mucosal	117.5 (28, 267.75)	.039
	Cutaneous	42 (20.5, 156.75)	
27	Oral mucosal	136 (36, 226)	.144
	Gastrointestinal	67 (19, 219)	
17	Oral mucosal	136 (53.5, 214)	.318
	Pulmonary	71 (27, 178.5)	
81	Xerostomia	144 (59, 305.5)	.001
	Cutaneous	64 (21, 166.5)	
46	Xerostomia	102 (35, 237)	.069
	Gastrointestinal	66 (12.25, 156)	
36	Xerostomia	113.5 (15.75, 283.5)	.839
	Pulmonary	91.5 (26.25, 250.5)	
31	Dysgeusia	145 (35, 357)	.200
	Cutaneous	64 (21, 229)	
20	Dysgeusia	170.5 (35, 404.5)	.255
	Gastrointestinal	104 (21.25, 213.75)	
11	Dysgeusia	65 (23, 245)	.896
	Pulmonary	71 (39, 161)	

In patients who developed an oral irAE and a non-oral irAE, the non-oral irAE generally developed earlier than the oral irAE. For patients with both oral mucosal and cutaneous irAEs (*n* = 46), the cutaneous symptoms presented significantly earlier onset (*P* = .039, Wilcoxon signed-rank test). Similar significantly earlier onset of cutaneous symptoms was also noticed in patients with both xerostomia and cutaneous irAEs (*n* = 81) (*P* = .001, Wilcoxon signed-rank test).

Abbreviations: irAE: immune-related adverse event; IQR: interquartile range.

### Characteristics of Matched Patients With and Without Oral irAEs

Forty-four patients with melanoma, 76 patients with lung cancer, and 33 patients with head and neck cancer patients with no oral irAEs were selected to compare the prevalence of non-oral irAEs in patients with versus without oral irAEs. Age, gender, ICIT regimen, and the number of infusions were matched at a 1:1 ratio. There was no statistical difference in the age, number of infusions, and total treatment duration days between the group of patients with oral irAEs versus the matched group of patients without oral irAEs. In patients with oral irAEs, 46 had oral mucosal disorders, 109 had xerostomia, and 27 had dysgeusia (Table 3).

**Table 3.** Characteristics and non-oral irAEs in the matched patients with and without oral irAEs.

Characteristics	Oral irAE (*n* = 153)	No oral irAE (*n* = 153)	OR (95% CI)	*P*
Age, years	67.1 ± 10.8	66.7 ± 10.4	—	.685
Female	58 (37.9)	58 (37.9)	—	—
Immune checkpoint inhibitor regimen (%)				
Pembrolizumab	69 (45.1)	69 (45.1)	—	—
Nivolumab	51 (33.3)	51 (33.3)	—	—
Ipilimumab + Nivolumab	16 (10.5)	16 (10.5)	—	—
Ipilimumab + Pembrolizumab	6 (3.9)	6 (3.9)	—	—
Atezumab	5 (3.3)	5 (3.3)	—	—
Ipilimumab + Nivolumab + Pembrolizumab	3 (2.0)	3 (2.0)	—	—
Other regimens	3 (2.0)	3 (2.0)	—	—
Number of infusions (IQR)	7 (2.5, 16)	7 (3, 15.5)	—	0.923
Treatment duration days (IQR)	147 (29, 363)	123 (28.5, 335)	—	0.633
Type of non-oral irAE (%)				
Mucosal disorder	46	—	—	—
Xerostomia	109	—	—	—
Dysgeusia	27	—	—	—
Type of non-oral irAE (%)			—	—
Any	104 (68.0)	83 (54.2)	1.79 (1.12, 2.85)	0.014
Cutaneous	53 (34.6)	36 (23.5)	1.72 (1.04, 2.84)	0.032
Gastrointestinal	34 (22.2)	30 (19.6)	1.17 (0.67, 2.03)	0.574
Pulmonary	25 (16.3)	20 (13.1)	1.30 (0.69, 2.45)	0.419
Rheumatological	23 (15.0)	13 (8.5)	1.91 (0.93, 3.92)	0.076
Thyroid	16 (10.5)	6 (3.9)	2.86 (1.09, 7.52)	0.027
Hepatic	11 (7.2)	9 (5.9)	1.24 (0.5, 3.08)	0.644
Neurologic	10 (6.5)	6 (3.9)	1.71 (0.61, 4.84)	0.304
Ocular	7 (4.6)	6 (3.9)	1.17 (0.39, 3.58)	0.777
Hematologic	3 (2.0)	4 (2.6)	0.75 (0.16, 3.39)	1.000^a^
Cardiac	4 (2.6)	2 (1.3)	2.03 (0.37, 11.23)	0.684^a^
Endocrine	5 (3.3)	1 (0.7)	5.14 (0.59, 44.48)	0.214^a^
Pituitary	5 (3.3)	0	—	0.061^a^
Renal	4 (2.6)	0	—	0.123^a^

^a^Fisher’s exact test.

Abbreviations: IQR: interquartile range; irAEs: immune-related adverse events.

### Correlation of Oral irAEs and Non-oral irAEs

**Figure 4.**
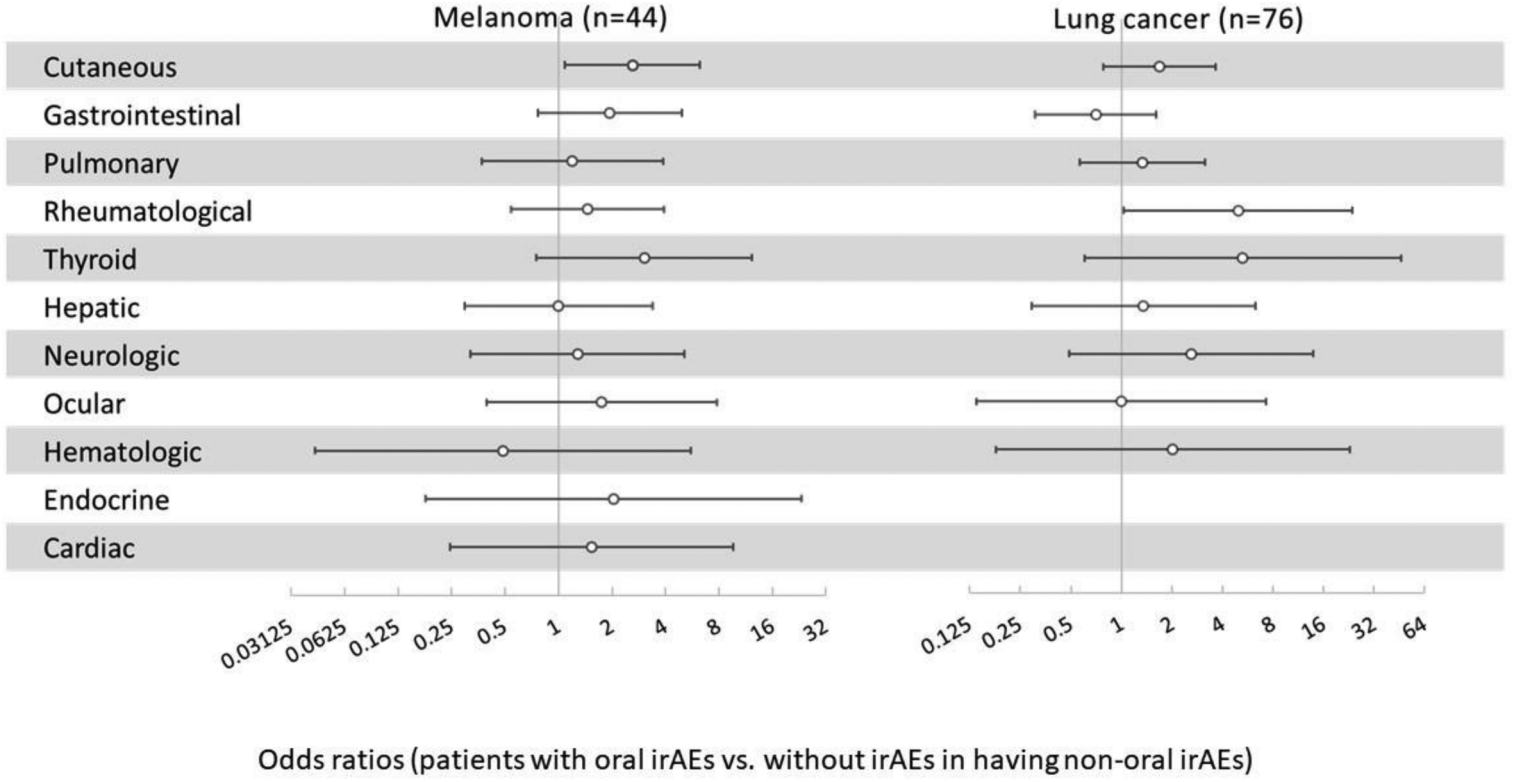
Association of oral irAEs and non-oral irAEs in patients with melanoma or lung cancer: Patients with melanoma (*n* = 44) and lung cancer (*n* = 76) with oral irAEs were matched with the same number of patients without oral irAEs as previously described. The risk of having a non-oral irAE associated with oral irAEs was assessed by the odds ratios (dot) with 95% confidence intervals (bar). Patients without oral irAEs were considered as the reference. Oral irAEs were associated with cutaneous irAEs in patients with melanoma and were associated with rheumatological irAEs in patients with lung cancer. Abbreviations: irAEs: immune-related adverse events.

The comparison of non-oral adverse events in the patients who had oral irAEs with the matched group of patients with no oral irAEs is listed in Table 3. Patients with oral irAEs were more likely to have cutaneous irAE (OR: 1.72, 95% CI, 1.04-2.84) and thyroid irAEs (OR: 2.86, 95% CI, 1.09-7.52). In the subgroups by the primary cancer diagnosis, patients with melanoma with oral irAEs were more likely to have cutaneous irAEs, (OR: 2.61, 95% CI, 1.09-6.27). In patients with lung cancer, those who had oral irAEs were more likely to have rheumatological irAEs (OR: 4.97, 95% CI, 1.04-23.8) (Fig. 4). In patients with head and neck cancer, no significant association of oral irAEs and non-oral irAEs was noticed. There was no significant difference regarding the days to onset of cutaneous, gastrointestinal, and pulmonary irAEs in patients with oral irAEs and matched patients without oral irAEs.

## Discussion

We comprehensively assessed non-oral irAEs in patients with oral irAEs using a large clinical dataset in a real-world setting. Among the 314 patients who experienced oral irAEs, 69.1% (*n* = 217) had at least one other non-oral irAE. In patients with oral mucosal irAEs (*n* = 106), 80.6% (*n* = 85) had other non-oral irAEs. This was higher than what was reported by Jacob et al., where 64% of patients with oral mucositis presented with other irAEs after the anti-CTLA-4 or anti-PD-1/PD-L1 treatment.^11^

Our study found that cutaneous, gastrointestinal, and rheumatological irAEs were the most common non-oral irAEs reported in patients, whether they had oral irAEs or not. This is consistent with the toxicity profiles reported previously, as the toxicities commonly affect the skin, gastrointestinal system, joints, and other organs.^3,12,13^ However, the previous incidence rates of irAEs were based mostly based on clinical trials, which could defer from real-world settings. For instance, the more severe irAEs leading to hospitalization are more likely to be documented and highlighted. Recent findings have shown that more than 20% of patients admitted for irAEs experience multiple toxicities, with gastrointestinal and cutaneous toxicities being two of the more common irAEs leading to hospitalization.^14^

Oral toxicities may be reported more frequently in patients admitted for more comprehensive management of their irAEs. Regarding rheumatological irAEs, the phenotype of inflammatory arthritis/arthralgias has been found to vary depending on the ICIT regimen.^15^ Rheumatological irAEs can have a slower, more discrete onset, leading to delayed reporting of symptoms, similar to oral irAEs.

Dermatological irAEs have been previously reported as one of the most common irAE with a wide range of clinical presentations,^16^ including morbilliform or lichenoid eruptions, vitiligo, pruritus, bullous disorders, and psoriasiform or eczematous dermatitis.^17,18^ It is not surprising that oral irAEs can occur concurrently with or be part of the cutaneous irAEs due to the similarity of oral mucosa and skin in architecture, function, and immune reaction patterns. Patients who developed lichenoid eruptions,^19^ bullous disorders,^20-22^ erythema multiforme,^23,24^ Stevens-Johnson syndromes,^25,26^ or toxic epidermal necrolysis^27^ were also commonly presented with oral symptoms, indicating the close correlation between the oral and cutaneous irAEs. The association of oral irAEs and cutaneous irAEs was further supported by our work. The incidence of cutaneous irAEs was significantly higher in those who experienced oral irAEs compared to those who did not in our matched analysis (*P* = .032). Also, the extent of oral irAEs was associated with the presence of cutaneous irAEs.

Gastrointestinal toxicities are also one of the most commonly reported irAEs.^28^ In our study, 17.7% of patients with oral mucosal irAEs had gastrointestinal symptoms, which is close to the concurrent incidence of gastrointestinal irAEs in patients with ICI-related oral mucositis (15.7%), as previously reported.^11^ However, no significant association between oral irAEs and gastrointestinal irAEs was found when comparing patients with oral irAEs and those without in the following analysis. Although it is thought that immune surveillance in lamina propria can affect both oral and gastrointestinal compartments as they are considered as the same immune continuum, recent studies have demonstrated the unique response in the oral cavity versus the gastrointestinal tract, which is due to the difference in epithelial structure, cell-to-cell junctions, microbial environment, and cell signaling.^28,29^

Regarding other non-oral irAEs, thyroid irAEs were significantly associated with oral irAEs. Rheumatological and renal irAEs were significantly associated with oral irAEs among patients with lung cancer, which indicates that oral irAEs can reflect both irAEs affecting the organ with extensive environmental interfaces (such as the skin) as well as the organs with presumed pre-existing autoimmunity (such as the thyroid and joints).

In 314 patients with oral irAEs, experiencing more than one oral irAE was significantly associated with higher odds of also experiencing at least one non-oral irAE (such as cutaneous, rheumatological, thyroid, and neurologic irAEs). Patients with multiple oral irAEs may present with various mechanisms leading to increased activated T cells, proinflammatory cytokines, enhanced complement activity, and autoantibody-mediated immune injury.^13,30^ The scope of irAEs is broad, with symptoms affecting virtually every organ system.

There is a lack of studies exploring the progression of irAEs and the temporal relation between non-oral and oral irAEs. Previous studies focused on using the development of systemic irAEs as a therapeutic marker and assessing their association with the therapeutic efficacy of ICIs in treating cancer.^31-33^ The trajectory analysis from our study revealed that, for the majority of patients who developed both oral and non-oral irAEs, non-oral developed within fewer days than oral irAEs (Table 2). While the pathophysiology of irAEs is still unclear, we propose three possible explanations for the temporal relationship between oral and non-oral irAEs. First, certain oral manifestations may not be symptomatic or be of mild severity, which might result in them not being recorded during the examination. Second, there is no standardized method for conducting oral examinations within the hospital setting, which could impact when oral symptoms are reported and addressed. Finally, oral irAEs, including mucosal disorders (involving epithelial injury) and xerostomia/dry mouth (related to salivary gland damage) could be associated with either cell-mediated or antibody-mediated immune responses, hence reflecting varied onset times of oral symptoms. In addition, the symptom profile for oral irAEs and non-oral irAEs falls on a spectrum and may also depend on cancer type.^34^

### Strengths and Limitations

To our knowledge, our study is the first to investigate the temporal relation between oral and non-oral irAEs in patients with cancer in a real-world setting. This approach enables us to explore irAEs in more diverse populations that may be underrepresented in clinical trials or may present with late-onset irAEs.^12^ We employed relevant statistical methods to generate control groups, adjusting for confounding factors such as age, gender, cancer type, and ICI type when comparing the incidence of non-oral irAEs. However, some heterogeneity still existed between the study and control groups due to our limited sample size. This study was a retrospective analysis involving significant chart review, which increases the risk of recall bias and multiple interpretations of EMRs. As has been consistently reported relative to cancer treatment side effects, a large difference exists in reported frequencies and severity characterizations between studies in which treatment toxicities are the primary objective compared with those in which the same toxicities are reported off-hand. For this reason, the severity of irAEs was not assessed, and there may be underreporting of adverse events associated with ICIT.

## Conclusions

In summary, we defined the associations of oral and non-oral irAEs and showed that patients with oral irAEs were at higher risk of having a non-oral irAE, and patients with multiple oral irAEs were more likely to have at least one non-oral irAEs compared to patients with a single oral irAE. ICIT treatment-related oral toxicities were associated with other non-oral toxicities and, therefore, required multidisciplinary care.

## Supplementary Material

Supplementary material is available at *The Oncologist* online.

oyad279_suppl_Supplementary_Material_S1

## Data Availability

The data that support the findings of this study are available from Mass General Brigham. Restrictions apply to the availability of these data, which were used under license for this study. Data are available from the authors with the permission of Mass General Brigham.

## Presented At

Data from an earlier cutoff date from this study were presented at 2022 American Academy of Oral Medicine annual meeting “Characteristics Of Oral Immune-Related Adverse Events (IrAEs) Of Immune Checkpoint Inhibitors And Their Correlation With Other Systemic IrAEs.”
