# Correction to: Comparisons of Nonoral Immune-Related Adverse Events Among Patients With Cancer With Different Oral Toxicity Profiles

**DOI:** 10.1093/oncolo/oyad318

**Published:** 2023-11-29

This is a correction to: Yuanming Xu, Natalie Wen, Robert I Haddad, Stephen T Sonis, Alessandro Villa, Comparisons of Nonoral Immune-Related Adverse Events Among Patients With Cancer With Different Oral Toxicity Profiles, *The Oncologist*, 2023;, oyad279, https://doi.org/10.1093/oncolo/oyad279

In the originally published version of this manuscript, there were errors in Table 3.

**Table 3.** Characteristics and nonoral irAEs in the matched patients with and without oral irAEs.

Characteristics	Oral irAE (*n* = 153)	No oral irAE (*n* = 153)	OR (95% CI)	*P*
Age, years	67.1 ± 10.8	66.7 ± 10.4	—	.685
Female	116 (37.9)	116 (37.9)	—	—
Immune checkpoint inhibitor regimen (%)				
Pembrolizumab	69 (45.1)	69 (45.1)	—	—
Nivolumab	51 (33.3)	51 (33.3)	—	—
Ipilimumab + Nivolumab	16 (10.4)	16 (10.5)	—	—
Ipilimumab + Pembrolizumab	6 (3.9)	6 (3.9)	—	—
Atezumab	5 (3.3)	5 (3.3)	—	—
Ipilimumab + Nivolumab + Pembrolizumab	3 (2.0)	3 (2.0)	—	—
Other regimens	3 (2.0)	3 (2.0)	—	—
Number of infusions (IQR)	7 (2.5, 16)	7 (3, 15.5)	—	.923
Treatment duration days (IQR)	147 (29, 363)	123 (28.5, 335)	—	.633
Type of nonoral irAE (%)				
Mucosal disorder	46	—	—	—
Xerostomia	109	—	—	—
Dysgeusia	27	—	—	—
Type of nonoral irAE (%)			—	—
Any	104 (54.2)	83 (68.0)	1.79 (1.12, 2.85)	.014
Cutaneous	53 (34.6)	36 (23.5)	1.72 (1.04, 2.84)	.032
Gastrointestinal	34 (22.2)	30 (19.6)	1.17 (0.67, 2.03)	.574
Pulmonary	25 (16.3)	20 (13.1)	1.3 (0.69, 2.45)	.419
Rheumatological	23 (15)	13 (8.5)	1.91 (0.93, 3.92)	.076
Thyroid	16 (10.5)	6 (3.9)	2.86 (1.09, 7.52)	.027
Hepatic	11 (7.2)	9 (5.9)	1.24 (0.5, 3.08)	.644
Neurologic	10 (6.5)	6 (3.9)	1.71 (0.61, 4.84)	.304
Ocular	7 (4.6)	6 (3.9)	1.17 (0.39, 3.58)	.777
Hematologic	3 (2)	4 (2.6)	0.75 (0.16, 3.39)	1.000^a^
Cardiac	4 (2.6)	2 (1.3)	2.03 (0.37, 11.23)	.684^a^
Endocrine	5 (3.3)	1 (0.7)	5.14 (0.59, 44.48)	.214^a^
Pituitary	5 (3.3)	0	—	.061^a^
Renal	4 (2.6)	0	—	.123^a^

^a^Fisher’s exact test.

Abbreviations: IQR: interquartile range; irAEs: immune related-adverse events.

Table 3 should read:

Table 3. Characteristics and nonoral irAEs in the matched patients with and without oral irAEs.

**Table:** 

Characteristics	Oral irAE (*n* = 153)	No oral irAE (*n* = 153)	OR (95% CI)	*P*
Age, years	67.1 ± 10.8	66.7 ± 10.4	—	.685
Female	58 (37.9)	58 (37.9)	—	—
Immune checkpoint inhibitor regimen (%)				
Pembrolizumab	69 (45.1)	69 (45.1)	—	—
Nivolumab	51 (33.3)	51 (33.3)	—	—
Ipilimumab + Nivolumab	16 (10.5)	16 (10.5)	—	—
Ipilimumab + Pembrolizumab	6 (3.9)	6 (3.9)	—	—
Atezumab	5 (3.3)	5 (3.3)	—	—
Ipilimumab + Nivolumab + Pembrolizumab	3 (2.0)	3 (2.0)	—	—
Other regimens	3 (2.0)	3 (2.0)	—	—
Number of infusions (IQR)	7 (2.5, 16)	7 (3, 15.5)	—	.923
Treatment duration days (IQR)	147 (29, 363)	123 (28.5, 335)	—	.633
Type of nonoral irAE (%)				
Mucosal disorder	46	—	—	—
Xerostomia	109	—	—	—
Dysgeusia	27	—	—	—
Type of nonoral irAE (%)			—	—
Any	104 (68.0)	83 (54.2)	1.79 (1.12, 2.85)	.014
Cutaneous	53 (34.6)	36 (23.5)	1.72 (1.04, 2.84)	.032
Gastrointestinal	34 (22.2)	30 (19.6)	1.17 (0.67, 2.03)	.574
Pulmonary	25 (16.3)	20 (13.1)	1.30 (0.69, 2.45)	.419
Rheumatological	23 (15.0)	13 (8.5)	1.91 (0.93, 3.92)	.076
Thyroid	16 (10.5)	6 (3.9)	2.86 (1.09, 7.52)	.027
Hepatic	11 (7.2)	9 (5.9)	1.24 (0.5, 3.08)	.644
Neurologic	10 (6.5)	6 (3.9)	1.71 (0.61, 4.84)	.304
Ocular	7 (4.6)	6 (3.9)	1.17 (0.39, 3.58)	.777
Hematologic	3 (2.0)	4 (2.6)	0.75 (0.16, 3.39)	1.000^a^
Cardiac	4 (2.6)	2 (1.3)	2.03 (0.37, 11.23)	.684^a^
Endocrine	5 (3.3)	1 (0.7)	5.14 (0.59, 44.48)	.214^a^
Pituitary	5 (3.3)	0	—	.061^a^
Renal	4 (2.6)	0	—	.123^a^

a Fisher’s exact test.

Abbreviations: IQR: interquartile range; irAEs: immune related-adverse events.

instead of:

There was also an error in Figure 1. The melanoma sample size should read: “44” instead of: “41”.

These errors have been corrected in the article.

