# Supplementary material for: Comparisons of Non-Oral Immune-Related Adverse Events Among Patients With Cancer With Different Oral Toxicity Profiles
Source: Oncologist. 2023 Oct 24;29(3):e382–91. doi: 10.1093/oncolo/oyad279 (PMC10911904; doi:10.1093/oncolo/oyad279)
Supplement: oyad279_suppl_Supplementary_Material_S1 [file oyad279_suppl_supplementary_material_s1.docx]

| **Supplement 1: Clinical characteristics of oral irAEs and associated non-oral irAEs**. | | | | | | | | |
| --- | --- | --- | --- | --- | --- | --- | --- | --- |
|  | Xerostomia only | Mucosal only | Dysgeusia only | Mucosal+  Xerostomia | Xerostomia+  Dysgeusia | Mucosal+  Dysgeusia | All three | Total |
| n | 155 | 60 | 33 | 24 | 20 | 6 | 16 | 314 |
| Dermatological | 53 (34.2) | 24 (40) | 10 (30.3) | 11 (45.8) | 10 (50.0) | 4 (66.7) | 7 (43.8) | 119 (37.9) |
| Gastrointestinal | 29 (18.7) | 14 (23.3) | 8 (24.2) | 7 (29.2) | 6 (30.0) | 2 (33.3) | 4 (25.0) | 70 (22.3) |
| Rheumatological | 22 (14.2) | 11 (18.3) | 3 (9.1) | 9 (37.5) | 5 (25.0) | 2 (33.3) | 2 (12.5) | 54 (17.2) |
| Pulmonary | 23 (14.8) | 6 (10.0) | 4 (12.1) | 7 (29.2) | 3 (15.0) | 1 (16.7) | 3 (18.8) | 47 (15.0) |
| Thyroid | 16 (10.3) | 5 (8.3) | 4 (12.1) | 6 (25.0) | 4 (20.0) | 0 (0) | 4 (25.0) | 39 (12.4) |
| Hepatic | 15 (9.7) | 11 (18.3) | 2 (6.1) | 0 (0) | 2 (10.0) | 0 (0) | 2 (12.5) | 32 (10.2) |
| Neurologic | 6 (3.9) | 6 (10) | 1 (3.0) | 3 (12.5) | 3 (15.0) | 0 (0) | 2 (12.5) | 21 (6.7) |
| Hematologic | 4 (2.6) | 3 (5) | 2 (6.1) | 1 (4.2) | 2 (10.0) | 0 (0) | 1 (6.3) | 13 (4.1) |
| Renal | 8 (5.2) | 1 (1.7) | 1 (3.0) | 2 (8.3) | 0 (0) | 0 (0) | 1 (6.3) | 13 (4.1) |
| Ocular | 5 (3.2) | 4 (6.7) | 0 (0) | 2 (8.3) | 1 (5.0) | 0 (0) | 1 (6.3) | 13 (4.1) |
| Endocrine | 8 (5.2) | 0 (0) | 0 (0) | 2 (8.3) | 0 (0) | 0 (0) | 0 (0) | 10 (3.2) |
| Pituitary | 5 (3.2) | 1 (1.7) | 0 (0) | 0 (0) | 1 (5.0) | 0 (0) | 0 (0) | 7 (2.2) |
| Cardiac | 3 (1.9) | 2 (3.3) | 0 (0) | 1 (4.2) | 0 (0) | 0 (0) | 1 (6.3) | 7 (2.2) |
| irAEs: immune-related adverse events | | | | | | | | |
